# Harnessing brain plasticity to improve binocular vision in amblyopia: An evidence-based update

**DOI:** 10.1177/11206721231187426

**Published:** 2023-07-10

**Authors:** Benjamin Thompson, Maria Concetta Morrone, Peter Bex, Anthony Lozama, Bernhard A. Sabel

**Affiliations:** 1 Department of Optometry and Vision Science, 8430 University of Waterloo, Waterloo, ON, Canada; 2 Centre for Eye and Vision Science, Hong Kong; 3 Department of Translational Research on New Technologies in Medicine and Surgery, 9310 University of Pisa, Pisa, Italy; 4 Department of Psychology, Northeastern University, Boston, MA, USA; 5 33412 Novartis Pharmaceutical Corporation, East Hanover, NJ, USA; 6 Institute of Medical Psychology, Faculty of Medicine, Otto-von-Guericke University of Magdeburg, Magdeburg, Germany

**Keywords:** Amblyopia, neuro ophthalmology, strabismus, eye movement disorders, Eso and Exo deviations

## Abstract

Amblyopia is a developmental visual disorder resulting from atypical binocular experience in early childhood that leads to abnormal visual cortex development and vision impairment. Recovery from amblyopia requires significant visual cortex neuroplasticity, i.e. the ability of the central nervous system and its synaptic connections to adapt their structure and function. There is a high level of neuroplasticity in early development and, historically, neuroplastic responses to changes in visual experience were thought to be restricted to a “critical period” in early life. However, as our review now shows, the evidence is growing that plasticity of the adult visual system can also be harnessed to improve vision in amblyopia. Amblyopia treatment involves correcting refractive error to ensure clear and equal retinal image formation in both eyes, then, if necessary, promoting the use of the amblyopic eye by hindering or reducing visual input from the better eye through patching or pharmacologic therapy. Early treatment in children can lead to visual acuity gains and the development of binocular vision in some cases; however, many children do not respond to treatment, and many adults with amblyopia have historically been untreated or undertreated. Here we review the current evidence on how dichoptic training can be used as a novel binocular therapeutic approach to facilitate visual processing of input from the amblyopic eye and can simultaneously engage both eyes in a training task that requires binocular integration. It is a novel and promising treatment for amblyopia in both children and adults.

## Introduction

Neuroplasticity is the ability of the central nervous system (CNS) and its synaptic connections to adapt to structural and functional demands and is critical for neurodevelopment, learning, and recovery from CNS damage. Visual system neuroplastic changes occur in response to visual experiences such as visual deprivation, environmental changes, and disrupted binocular vision.^1^ In early development, there is a high level of neuroplasticity. Historically, based on observations from animal studies, neuroplastic responses to changes in visual experience were thought to be restricted to a period early in life, called the “critical period”^2,3^; this is why amblyopia is often considered treatable in children but not, or less so, in adults.^4^ However, it is now established that the degree of neuroplasticity is greater in the adult visual system than previously thought; this provides new opportunities for visual neurorehabilitation and restoration far beyond the critical period.^5–9^

Amblyopia is a developmental visual disorder resulting from atypical binocular experience in early childhood that causes abnormal visual cortex development and vision impairment.^4,10^ Recovery from amblyopia relies on significant visual cortex neuroplasticity. Treatment involves correcting refractive error to ensure clear and equal retinal images in both eyes, then, if necessary, promoting (or forcing) the use of the amblyopic eye by hindering or blocking visual signaling to the brain from the better eye (e.g. patching and pharmacologic therapy).^11^ Early treatment in children can lead to considerable acuity gains in the affected eye(s) and, in some cases, to the development of binocular vision and depth perception. However, current approaches are limited, as many children may fail to adhere to the patching schedule and do not respond to treatment; furthermore, adults with amblyopia are typically not treated as they are outside the critical period.^4,12^

Because our understanding of visual system plasticity has made much progress in recent years, here we describe the current understanding of neuroplasticity in human amblyopia, including developmental and adult mechanisms of plasticity, current treatments, and the potential benefits of dichoptic stimulation to improve binocular vision in amblyopia.

## Current understanding of binocular vision plasticity in amblyopia

### Causes of amblyopia

The major types of amblyopia are strabismic, anisometropic, combined strabismic and anisometropic, ametropic, and deprivation (Table 1),^11^ but the degree of functional impairment and potential for the development of binocular vision vary considerably. Strabismus, anisometropia, and ametropia likely affect visual cortex development differently. While strabismus has the greatest impact on binocular visual development, individuals with anisometropic amblyopia often still have some binocular vision.^11,13–15^ Patients with intermittent strabismus may be easier to treat than those with strabismic (or constant strabismic) amblyopia, as they have occasional ocular alignment and activation of binocular neural circuits.^15^

**Table 1.** Major types of amblyopia.^11^

Type	Description	Example (Top: left eye - right eye; Bottom: Combined visual experience)
Strabismic	Misalignment of eyes (“squinting”) results in mismatched/decorrelated neural inputs to the visual cortex that lead to interocular suppression and unilateral amblyopia.	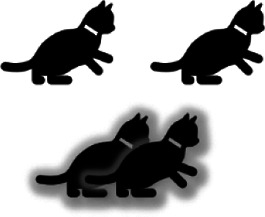
Anisometropic	A difference in refractive error between both eyes (typically hyperopia or astigmatism) leads to chronic blurring of the retinal image in one eye. This causes a difference in the contrast and spatial frequency content of the inputs from each eye to the visual cortex resulting in interocular suppression and unilateral amblyopia.	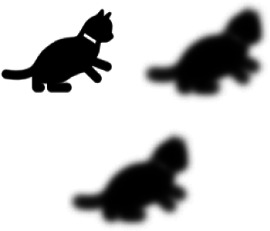
Combined strabismic and anisometropic	Eye misalignment and a blurred image in one eye (typically the deviated eye) occur simultaneously and cause interocular suppression and unilateral amblyopia.	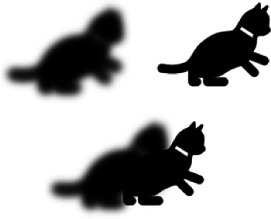
Ametropic	Bilateral, symmetric high refractive error resulting in blurred vision in both eyes causes amblyopia in both eyes.	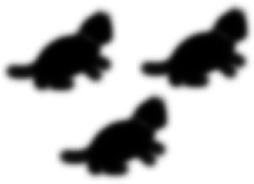
Deprivation	Obstruction of the visual axis results in impaired development of the visual cortex; it may be bilateral or unilateral.	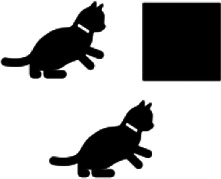

### The critical period and plasticity in amblyopia

Disrupted visual input to the brain during an early “critical” or “sensitive period” of heightened neuroplasticity induces structural and functional changes within neural systems.^7^ Animal models of unilateral amblyopia have been used extensively to explore this period of mammalian visual development.^7,16^ Historically, monocular deprivation was the most common technique used to induce amblyopia in animals. In their ground-breaking, Nobel-prize-winning work, Hubel and Wiesel found that a short, 3-month period of unilateral deprivation in kittens could cause lifelong vision impairment.^6^ Furthermore, monocular deprivation for 1 month in kittens as young as 9 weeks led to a larger number of cortical cells strongly preferring the non-deprived eye versus the deprived eye. In contrast, adult cats showed no cortical deficits following monocular deprivation, thus indicating that young animals are more susceptible to visual deprivation than mature animals.^6^

**Figure 1.**
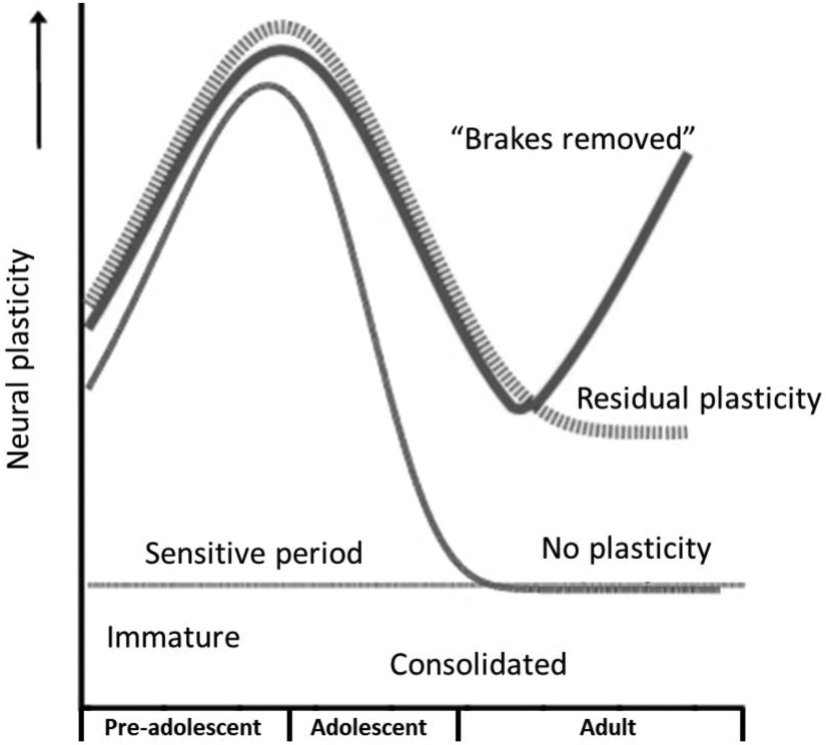
The sensitive period of neural plasticity during development. The lower light grey curve shows the traditional concept of developmental plasticity, which is very high during early development when the neural system is still immature, peaking, and rapidly diminishing to zero when normal visual function is consolidated. The dashed curve shows how residual plasticity can extend well into adulthood, though at a lower level. The upper dark grey curve illustrates removing the factors (brakes) that limit plasticity and restoring levels of neural plasticity in adults. Figure reproduced with permission from Levi DM. *Vision Res* 2020;176:118–129. Copyright 2022, Elsevier.^17^

Given the different etiologies and types of amblyopia, multiple hypotheses exist about the temporal windows for neural plasticity in different visual areas throughout development (Figure 1). The idea that amblyopia in humans cannot be treated past childhood (around 7 or 8 years) is based on the concept that treatment for amblyopia is only effective during the early critical period for visual development when brain plasticity is still high. However, more recent studies in humans and nonhuman primates suggest that, rather than an abrupt and complete drop in plasticity with advancing age, there is considerable residual plasticity even in adulthood.^17^ In addition, there is evidence from mouse studies that it is possible to remove the “brakes” that limit neuroplasticity in the adult visual cortex.^4,17^

### Mechanisms of plasticity in amblyopia

**Figure 2.**
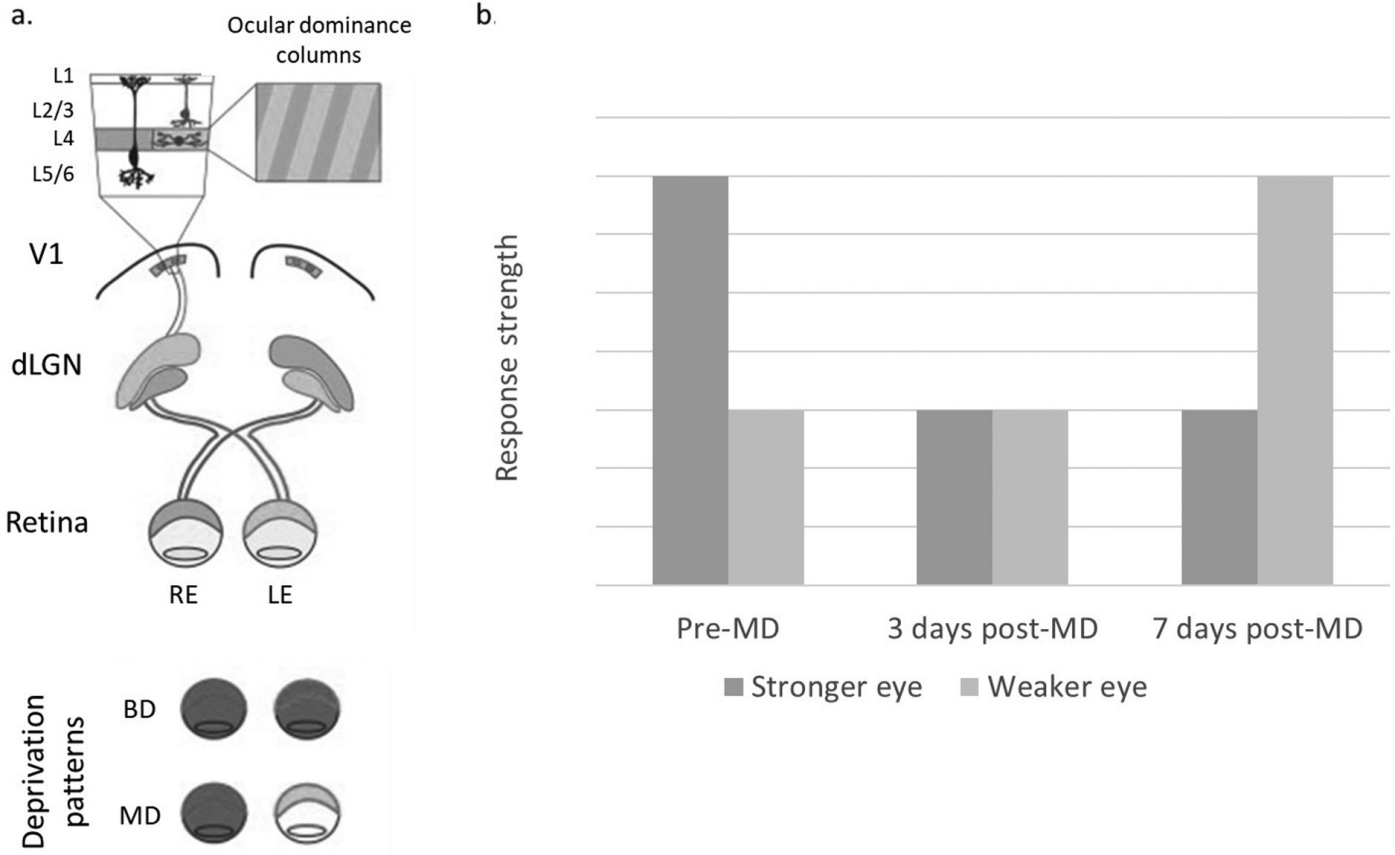
(a) Schematic showing the basic arrangement of the visual pathway in mammals. The visual and somatosensory systems are suitable targets for investigating the role of sensory experience in the regulation of the development and plasticity of neural circuits; activity along these sensory pathways can be manipulated, and this has historically been studied by raising animals in the dark and by depriving one (monocular deprivation) or both (binocular deprivation) eyes of patterned vision. Reproduced with permission from Fox K, Wong ROL. *Neuron* 2005;48:465–477. Copyright 2022, Cell Press^24^; (b) Physiological measurements of plasticity at thalamocortical synapses (e.g. layer 4 visually evoked potentials) of the mouse reveal two sequential changes; deprived-eye depression after 3 days of monocular deprivation and open-eye potentiation after 7 days of MD (darker = contralateral, stronger eye; lighter = ipsilateral, weaker eye). The figure also illustrates the ocular dominance of the stronger eye vs. the weaker eye present in amblyopia before treatment. Adapted from Coleman J, et al. The molecular and structural basis of amblyopia; In: The new visual neurosciences. 2013. p1433–1442.^25^ BD, binocular deprivation; dLGN, dorsal lateral geniculate nucleus; L, layer; LE, left eye; MD, monocular deprivation; RE, right eye; V1, primary visual cortex.

There are two major forms of plasticity in the CNS, “Hebbian” and “homeostatic” plasticity. Both play a role in the mechanisms of amblyopia and impact the design of novel treatments. Hebbian plasticity provides a synaptic basis for associative learning, where correlated activation of pre- and postsynaptic neurons strengthens the connection between both neurons (i.e. “what fires together, wires together”). In contrast, homeostatic plasticity stabilizes network firing rate in response to input activity, where the goal is to maintain stable input-output relation with neuronal firing rate as a readout.^17–20^ The Bienenstock–Cooper–Munro model is a modified version of the Hebbian model in which synaptic strength can be modified bidirectionally to promote synaptic weakening as well as strengthening with the threshold change in synaptic input, depending on the history of postsynaptic activity.^18,21^ In the ‘competition model’ of plasticity, the presence or absence of competitive input determines the mode of plasticity. Competition between a deprived (weaker) input and a spared (stronger) input typically triggers Hebbian plasticity, which either further depresses the weaker input and/or further strengthens the stronger input.^18^ For example, long-term monocular deprivation (if initiated early and continued through to the end of the critical period) induces a shift in ocular dominance. This is characterized by the shrinkage of ocular dominance columns in the visual cortex receiving signals from the deprived eye, leading to reduced visual acuity that is highly resistant to reversal (Figure 2).^5,22–25^

Homeostatic plasticity coexists with Hebbian neuroplasticity.^26,27^ Animal studies of Mrsic-Floge et al. show that *both* regulate neural activity during ocular dominance plasticity in early development.^19^ They showed that short periods of monocular deprivation reduced neuronal responses in the deprived eye in the mouse visual cortex. However, more extended periods of monocular deprivation strengthened both open-eye and deprived-eye responses. This observation of bidirectional response suggested that both Hebbian and homeostatic mechanisms regulate neuronal responses during experience-dependent plasticity.^19^

Monocular deprivation in adults for a few hours can boost the deprived eye signal, shifting ocular dominance in favor of the deprived eye.^28–31^ This counterintuitive effect of monocular deprivation reflects a compensatory reaction of the visual cortex to deprivation to keep the average cortical activity constant, indicating that some form of homeostatic plasticity is also present in adult humans.^18–20^ This plasticity is also retained in patients with amblyopia, both for the healthy fellow eye and the amblyopic eye, suggesting that inverse occlusion can potentiate the amblyopic eye signal, although it should be noted that this method is still experimental.^32–34^

In addition to Hebbian plasticity, homeostatic plasticity has been shown to operate during occlusion therapy for moderate amblyopia in children.^32^ Furthermore, a study of adults with amblyopia (N = 10) who underwent occlusion of the amblyopic eye combined with physical exercise showed that visual acuity improved in all patients after six brief 2-h training sessions, with six patients also recovering stereopsis.^34^

There is also evidence of independent monocular and binocular motor plasticity and dissociable spatial mapping for each eye.^35^ Binocular motor adaptation involves multiple, potentially competing processes: independent disconjugate adaptation of monocular saccades (rapid movement of each eye in opposite directions), conjugate adaptation of binocular saccades (rapid eye movement of both eyes in the same direction), and binocular adaptation of vergence eye movements.^35^ Also, amblyopic eyes show fewer fixational saccadic eye movements (microsaccades) than healthy control eyes.^36^ Microsaccades are well preserved throughout life; they are essential for high-resolution vision by synchronizing brain electrophysiological activity, and they can be impaired not only in amblyopia but also in other neurodegenerative or neuro-ophthalmologic diseases such as stroke.^37^

### Animal models harnessing plasticity to treat amblyopia

Several interventions have been identified in animals with amblyopia to recover vision in post-sensitive period animals.^7,38^ These include the chronic administration of the selective serotonin reuptake inhibitor fluoxetine,^8^ complete visual deprivation through dark exposure, exposure to enriched environments, temporary retinal inactivation, and exercise.^7,38^ Common mechanisms identified for some of these plasticity-promoting interventions include a reduction in neural inhibition within the visual cortex.^7^

### Is visual cortex plasticity possible in adult humans?

The neurophysiological basis of amblyopia in humans is not fully understood, but abnormal structure, connectivity, and function in a range of visual brain areas have been observed using magnetic resonance imaging.^7^ A review of current evidence suggests considerable plasticity is preserved well beyond the closure of the clinical periods for vision. One convincing example is that visual perception in adults with amblyopia can be improved in some patients through triggering homeostatic plasticity.^9^ The most notable cases of plasticity in adults with amblyopia are in the situation when the normal (non-amblyopic) eye is lost either through disease or injury: the vision can recover in the amblyopic eye – a clear sign that synapse formation and plasticity potential are preserved in the adult visual cortex.^17^ Some of the limits of plasticity in adults are structural, e.g. perineuronal nets or myelin, which inhibit neurite outgrowth. While a child's brain has developed the major connections from the eye to the brain, it is the synapses that determine if contact is established with downstream visual processing centers. These contacts are established if the connections are functionally used during visual processing, but if they are not used, synapses are not formed or are retracted. However, if used again (e.g. by visual training), synapses can form again (“use it or lose it”). Other contacts are functional, acting on the balance of excitation and inhibition within local neural circuits.^4^

### Variables influencing plasticity: Is age the only factor?

The efficiency of amblyopia treatment generally declines with age, but age alone does not explain outcome variability.^39^ Especially when testing younger children, there are variables related to the testing conditions to be considered when evaluating the effectiveness of treatment for improving VA: false positives are common when testing visual acuity in younger children; only a third of 3-year-old patients and half of 4-year-old patients can successfully complete a visual acuity test in each eye. Measurements may be more accurate in older children because the ability to complete visual acuity tests stabilizes over time.^40^

High variability in treatment response suggests there are factors other than age that can impact the success of therapies for amblyopia. One factor is that the level of improvement in amblyopia may depend on baseline ocular dominance.^41^ Ooi et al. demonstrated a ‘push-pull’ technique that forces total perceptual suppression of the strong eye while promoting excitatory signals in the weak eye.^42^ There is variation in responses to patching, and it is not currently possible to predict which patients will improve.^17^ Furthermore, adult plasticity is influenced by stress hormones,^43,44^ and recovery of vision is lower in patients who have personalities with low stress resilience.^43^ Therefore relaxed treatment environments may be beneficial. Homeostatic plasticity is more resilient to age, and by acting on homeostatic plasticity through reverse patching (patching the amblyopic eye), visual acuity can be recovered quickly in adults.^17^ Individual variability in treatment response outweighs age-related changes, for patching at least; studies on monitored occlusion show substantial variability within and between participants.^45^

In children with amblyopia, having some measurable stereopsis (vs. having none) may influence treatment outcomes. Children with no measurable stereopsis have a >2-fold increased risk for persistent amblyopia.^13,46^ Some recent studies have focused on direct training of stereopsis in individuals with amblyopia, with some success via perceptual learning, video games, and viewing 3D movies.^47–51^

## Status of amblyopia treatments

Based on evidence from animal models showing that the sensitive period for visual cortex plasticity can be manipulated and reopened to enable recovery of vision in animals beyond the early sensitive period, a growing number of studies are exploring novel techniques for treating amblyopia not only early in life but also in adulthood (Table 2).^7, 11, 14, 17, 21, 52–69^

**Table 2.** Principles of amblyopia therapies.

Amblyopia therapy	Key principles
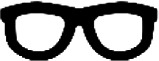 Refractive correction	Optical correction of any refractive error aims to ensure a clearly focused retinal image in each eye^17^ In children 3–7 years of age with no previous treatment, refractive correction alone improves visual acuity in many cases of anisometropic amblyopia and may resolve amblyopia^52^ Outcomes depends on the type of amblyopia, duration of therapy, and adherence to glasses wear^53^ Preliminary evidence suggests that refractive correction may also improve vision in some adults with amblyopia.^54,55^
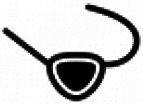 Occlusion therapy	Occlusion therapies include full- or part-time patching of the dominant eye to promote (force) the use of the amblyopic eye^56^ There are limitations to patching; 25% of children regress once the patch is removed,^21^ and patching is commonly considered ineffective after 10 years of age^57^ Patching can still be effective in previously untreated older children (13–17 years of age)^58^
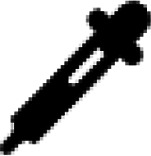 Atropine blurring	Instilling 1% atropine eye drops into the dominant eye blurs its visual acuity and promotes amblyopic eye use^11^ Treatment adherence may be higher for atropine blurring therapy than occlusion therapy.^58,59^ A study of atropine blurring vs. patching noted that although atropine was better accepted than patching, both treatments resulted in similar improvements in visual acuity.^59^
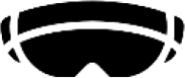 Binocular therapy	Although amblyopia is often considered a monocular disease,^46^ binocular dysfunction plays a vital role in amblyopia^46^ New treatment approaches (e.g. novel dichoptic training approaches), were developed based on a new understanding that humans with amblyopia can have latent binocular capabilities^46^ There have been mixed findings regarding the advantages of binocular therapy^14,41,48,59–68^ Further research is warranted to inform decisions about the implementation of binocular therapy for amblyopia in clinical practice^69^

### Refractive correction

Before the development of newer treatments, the main approach to the treatment of amblyopia was optical correction of any refractive error, with the aim of ensuring a clearly focused retinal image in each eye.^17^ In children 3–7 years of age with no previous treatment, refractive correction alone improves visual acuity in many cases of anisometropic amblyopia and may resolve amblyopia.^52^ However, the outcome is highly variable and depends on the type of amblyopia, duration of therapy, and adherence to glasses wear.^53^ Preliminary evidence suggests that refractive correction may also improve vision in some adults with amblyopia.^54,55^

### Occlusion therapy

Historically, most human studies of amblyopia have focused on unilateral amblyopia, and current treatments, therefore, target mainly monocular deficits in amblyopia. The primary treatment for unilateral amblyopia since the eighteenth century involves full- or part-time patching of the dominant eye to promote (force) the use of the amblyopic eye.^56^ The time needed for patching to be effective differs by the level of severity of amblyopia; it has been shown that for severe amblyopia, 6 h of patching a day can be effective as full-time patching,^70^ and for moderate amblyopia, 2 h of patching a day can be as effective as 6 h.^71^ More patients with anisometropic amblyopia than strabismic amblyopia show improvements in stereoacuity following patching.^13^

There are limitations to patching; 25% of children regress once the patch is removed,^21^ and patching is commonly considered ineffective after 10 years of age,^57^ yet patching can still be effective in previously untreated older children (13–17 years of age).^58^ The efficacy of patching treatments may be impacted by low adherence rates.^59^ In addition, it should be noted that adherence may be higher when shorter periods of patching are used, for example, in patients with severe amblyopia, ‘excellent’ patching was described for 53% of patients in the 6-h patching group vs. 32% of patients in the full-time patching group.^70^

### Atropine blurring therapy

Instilling 1% atropine eye drops into the dominant eye blurs its visual acuity and promotes amblyopic eye use.^11^ Treatment adherence may be higher for atropine blurring therapy than occlusion therapy.^72,73^ However, a study of atropine blurring vs. patching noted that although atropine was better accepted than patching, both treatments resulted in similar improvements in visual acuity.^73^ In children with moderate amblyopia, atropine blurring given on 2 consecutive days per week is as effective as daily use, and daily atropine is as effective at improving visual acuity as daily patching occlusion therapy in those with moderate amblyopia.^11,74,75^

### Perceptual learning and motor learning

Perceptual learning is a phenomenon whereby the repeated practice of a well-defined psychophysical task leads to improved task performance. Since it is robust and repeatable, it is an influential model for investigating neuroplasticity and the neural basis of sensory learning and visual cognition.^7,76–78^ Most perceptual learning studies in adults with amblyopia have involved patching the fellow eye and intensively training the participant on a psychophysical task while using their amblyopic eye, i.e. monocular perceptual training.^79,80^ Monocular perceptual training has been shown to substantially improve performance on a Vernier acuity task, and those improvements were also transferred to the non-trained eye.^80^ Subsequent studies, including randomized controlled studies and case studies, have demonstrated that monocular perceptual learning for various tasks, including contrast detection and discrimination of crowded targets, improved task performance, although the sample size in reported studies is generally small (N = 1–77).^7,57,79,81,82^

**Figure 3.**
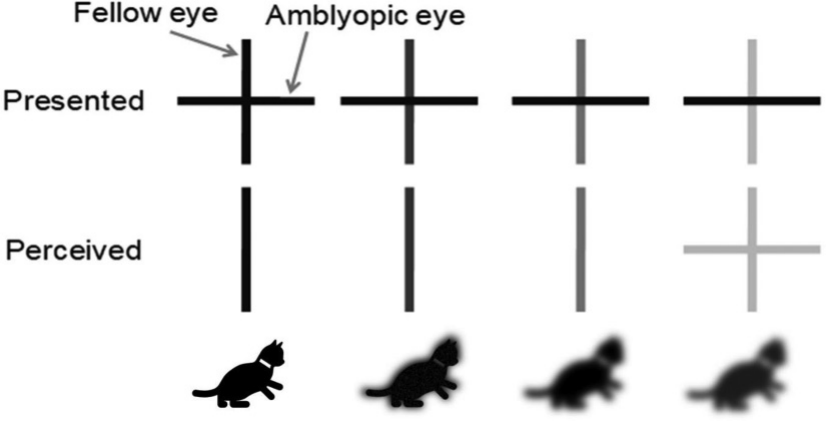
A schematic of the principle underlying dichoptic display therapy.^95^ As the contrast of the stimulus presented to the fellow eye is reduced, suppression of the amblyopia eye is reduced. Eventually, simultaneous perception of both eyes is achieved (bottom right).

Binocular perceptual learning techniques aim at overcoming barriers to binocular function in amblyopia, such as interocular suppression of the amblyopic eye and abnormal interocular attention^21,83,84^; the neural structures required to support binocular vision are present in amblyopia but not functional because of interocular suppression.^83,85,86^ There are conflicting findings as to whether dichoptic treatment reduces suppression, for example, a balanced binocular viewing therapy for amblyopia demonstrated rapid and substantial benefits that cannot be solely mediated by a reduction in interocular suppression.^87^ However, it is thought that interventions that directly target suppressive interactions within the visual cortex may be particularly relevant to the treatment of amblyopia.^83^ Methods to assess interocular imbalance in normal or amblyopic vision include binocular rivalry, dichoptic global motion coherence/orientation, binocular phase, and contrast combination.^85,88–94^ Binocular therapy is of particular interest because, unlike monocular approaches, it does not simply aim at strengthening the weak eye to become more active but it provides an opportunity for both eyes to learn how to work together. Binocular dichoptic contrast balancing therapy is based on the theory that the amblyopic visual system retains the capacity for binocular function and that binocular fusion can be improved (Figure 3).^93,95^

#### Principle of binocular therapy in amblyopia

Because amblyopia typically affects visual acuity in one eye, amblyopia is often considered a monocular disease, and treatments have focused on improving the monocular function of the amblyopic eye.^46^ However, binocular dysfunction plays a vital role in amblyopia, and new treatment approaches were developed to correct this problem.^46^ For example, novel dichoptic training approaches were proposed based on a new understanding that humans with amblyopia can have latent binocular capabilities. This overcomes traditional concepts that amblyopia treatments should only focus on monocular vision.^21^

## Dichoptic training as binocular therapy for amblyopia

### What is dichoptic training, and how does it harness plasticity?

Dichoptic training involves the presentation of different stimuli to each eye, as illustrated in Figure 3. It requires the summation and integration of two different stimuli presented to each eye to complete the dichoptic task successfully.^96^ In this manner, dichoptic training aims to improve binocular visual function. While one stimulus (image) is presented to the amblyopic eye (e.g. a bird), the fellow eye is shown an image that needs to be matched (e.g. a nest on a tree), so both can be fuzed by the brain (e.g. a bird sitting in the nest). This contrasts with typical perceptual learning tasks in which a single identical stimulus is presented alone to each eye.^96^

Dichotic training may act by balancing neuronal activity and integration, facilitating gains in vision,^21^ and its principles are based on Hebbian plasticity: the longer the visual system can be kept in a state where the presynaptic activity of both eyes correlates with postsynaptic activity, the more robust, permanent, and balanced will be the binocular integration and 3D vision.^21^ If interocular contrast is suitably adjusted to compensate for the amblyopic eye contrast threshold deficit, binocular summation at the threshold becomes normal.^86^ Individuals with strabismic and anisometropic amblyopia can have normal binocular function at specially selected interocular contrasts.^86^ Repeated exposure to binocularly balanced stimuli allows fusion to occur at increasingly smaller interocular contrast differences.^21^

### How does binocular therapy differ from patching of the amblyopic eye?

Patching is a short-term monocular therapy involving deprivation of the healthy eye to facilitate cortical processing of inputs from the amblyopic eye. In contrast, binocular therapy using contrast balancing is true dichoptic therapy, enabling simultaneous use of both eyes. With this approach, the primary aim is to restore binocular fusion and stereopsis.^21^

Previous treatments such as patching the non-amblyopic eye are ‘passive’ treatments in that they do not involve the patient's physical activity nor specifically train the integration of both eyes to work together. *Active* treatment approaches that require activity or a task for the patient are expected to be more beneficial than *passive* treatment approaches, particularly as the patient may be more likely to be compliant if the treatment is enjoyable, which increases the patient's motivation and attention.^97^ Advantages of binocular therapy include sensory-motor integration and cognitive effort or neuronal ‘load’. It may be more demanding on attention, enhancing plasticity.^98^ Active approaches to vision training have successfully restored vision and are well established in other diseases of partial blindness caused by visual system disorders such as glaucoma, optic nerve damage, or stroke,^99–103^ which are more effective when combined with attention tasks.^104^

### What clinical advantage does binocular therapy confer over monocular approaches?

There have been mixed findings regarding the advantages of binocular therapy. While visual outcomes (e.g. VA) are significantly improved with binocular games, alone or versus comparators,^14,41,48,60–65^ a systematic review and meta-analysis of randomized, controlled trials of vision-based treatments for children and teenagers with amblyopia showed no difference in treatment efficacy between treatments.^59,65^ Some studies of binocular games have not shown improved visual outcomes compared with placebo, spectacle correction, or patching.^66–68^ A Cochrane review of available data of conventional patching vs. novel binocular therapy concluded that it is not yet possible to draw robust conclusions regarding the overall safety and sustained effectiveness of binocular treatment, and further research is warranted to inform decisions about the implementation of binocular therapy for amblyopia in clinical practice.^69^

There are several video game-based systems currently available for the treatment of amblyopia, including *Dig Rush*,^60,68,105^ *Vivid Vision*,^106^ *Luminopia One*,^62^ and *BinoVision*.^107^ Of these, only *Dig Rush* and *Luminopia One* have been assessed in controlled randomized clinical trials. *Dig Rush* has produced superior visual acuity outcomes to patching in some trials and minimal effects in others, perhaps due to low adherence in some studies.^60,68,105^ *Luminopia One* (commercially available passive dichoptic videos/movie with head-mounted systems), has generated improvements in mean VA in children.^60,105^ In addition, a review of commercially available smartphone applications for amblyopia available on Android and iOS showed that most (93%) do not have eye care professional input, highlighting the need for stringent quality assurance of game-based treatments for amblyopia by qualified eye care professionals.^108^

It is important to note that adherence rates vary, and the advantages observed with binocular therapy may be due to gamification and the active achievement of increasing compliance in specific patient groups (e.g. older children).^59,66,109^ In addition, in the BRAVO study,^66^ adherence to home-based videogame treatment was characterized by short sessions interspersed with frequent pauses, suggesting regular disengagement. Gao et al. suggest that this can complicate dose-response calculations and interfere with the effectiveness of treatments like binocular treatments for amblyopia, which require sustained visual stimulation.^109^

### How can we design binocular therapy to maximize plasticity?

Several studies are now available that demonstrate visual acuity improvements in the amblyopic eye using dichoptic therapy.^62,64,110^ They show several ways in which binocular treatment could be tailored to the patient and which factors influence the outcome. The optimal duration of binocular therapy is likely age-dependent; in the PEDIG studies, fewer hours per day were required for younger subjects to achieve visual improvement.^63,68^ One hour per day of total treatment may be optimal, but therapy should be personalized for each patient.^68^ In a study of pre-school-aged children (N = 50), those who played binocular iPad games for ≥8 h over the course of 4 weeks had significantly more visual acuity gains than those who played for 0–4 h.^63^ In addition, children with moderate amblyopia had greater visual acuity improvement with a binocular game than those with severe amblyopia, and those who spent more time playing had more improvement.^41^ Of note, it is still difficult to know the precise dose and whether it was delivered continuously or in smaller bursts without adherence monitoring systems.

## Conclusions

The evidence is growing that the plasticity of the adult visual system can be harnessed in amblyopia to achieve gains in VA. Dichoptic training, a novel binocular therapeutic approach, is promising, as it facilitates input from the amblyopic eye while simultaneously engaging both eyes in a training task that requires binocular integration. In this way, amblyopia treatment is not just overcoming the dominance of the non-amblyopic eye but promotes binocular processing and integration to enable stereopsis. Especially if this therapy requires active engagement by the child, this may prove advantageous as it motivates the patient to comply, which may lead to superior outcomes, regardless of age. While the treatment needs to be tailored toward the individual needs of the patient, the type of amblyopia, previous treatment history, and age, such customized binocular (dichoptic) training is expected to harness neuroplasticity to maximize the potential of enabling binocular vision far beyond what can be accomplished with monocular therapies. Further exploration of dichoptic training for both children and adults is warranted to examine the ability to improve visual outcomes and gain a better understanding of the role of brain plasticity in vision restoration.
